# The burden of non communicable diseases in developing countries

**DOI:** 10.1186/1475-9276-4-2

**Published:** 2005-01-14

**Authors:** Abdesslam Boutayeb, Saber Boutayeb

**Affiliations:** 1 Department of Mathematical Sciences, Brunel University, Uxbridge, Middx UB8 3PH, UK; 2 Department of Mathematics, Faculty of Sciences, University Mohamed Ier, Oujda, Morocco; 3 Service Oncologie Médicale, Institut National d'Oncologie, Rabat, Morocco

## Abstract

**Background:**

By the dawn of the third millennium, non communicable diseases are sweeping the entire globe, with an increasing trend in developing countries where, the transition imposes more constraints to deal with the double burden of infective and non-infective diseases in a poor environment characterised by ill-health systems. By 2020, it is predicted that these diseases will be causing seven out of every 10 deaths in developing countries. Many of the non communicable diseases can be prevented by tackling associated risk factors.

**Methods:**

Data from national registries and international organisms are collected, compared and analyzed. The focus is made on the growing burden of non communicable diseases in developing countries.

**Results:**

Among non communicable diseases, special attention is devoted to cardiovascular diseases, diabetes, cancer and chronic pulmonary diseases. Their burden is affecting countries worldwide but with a growing trend in developing countries. Preventive strategies must take into account the growing trend of risk factors correlated to these diseases.

**Conclusion:**

Non communicable diseases are more and more prevalent in developing countries where they double the burden of infective diseases. If the present trend is maintained, the health systems in low-and middle-income countries will be unable to support the burden of disease. Prominent causes for heart disease, diabetes, cancer and pulmonary diseases can be prevented but urgent (preventive) actions are needed and efficient strategies should deal seriously with risk factors like smoking, alcohol, physical inactivity and western diet.

## Background

For centuries, communicable diseases were the main causes of death around the world. Life expectancy was often limited by uncontrolled epidemics. After the second World War, with medical research achievements in terms of vaccination, antibiotics and improvement of life conditions, non communicable diseases(NCDs) started causing major problems in industrialized countries. Heart diseases, cancer, diabetes, chronic pulmonary and mental diseases became a real burden for health systems in developed countries. For a while, these diseases were associated with economic development and so called diseases of the rich. Then, by the dawn of the third millennium, NCDs appeared sweeping the entire globe, with an increasing trend in developing countries (Table 1) where, the transition imposes more constraints to deal with the double burden of infective and non-infective diseases in a poor environment characterized by ill-health systems. In 1990 the leading causes of disease burden were pneumonia, diarrhoeal diseases and perinatal conditions. By 2020, it is predicted that NCDs will account for 80 percent of the global burden of disease, causing seven out of every 10 deaths in developing countries, compared with less than half today[1,2].

**Table 1:** Evolution of NCDs in developing countries (in million) [1,8,9]

	Non-Communicable Diseases	Communicable Diseases + Maternal + Perinatal + Nutritional	Injuries	total
1990	18.7 (47%)	16.6 (42%)	4.2 (11%)	39.5 (100%)
2000	25.0 (56%)	14.6 (33%)	5.0 (11%)	45.0 (100%)
2020	36.6 (69%)	09.0 (17%)	7.4 (14%)	53.0 (100%)

Efficient (preventive)strategies are needed and urgent measures should be taken to control risk factors like tobacco, alcohol, obesity, blood pressure diet and inactivity. Otherwise, developing countries will be unable to provide their people with standard health care.

The costly and prolonged treatment of NCDs raises the equity problem between and within countries. As expressed by the WHO Director-General in his overview to the annual report[1], If a Japanese woman develop chronic diseases, excellent treatment and rehabilitation services will be available and she can expect to receive, on average, medications worth about US$ 550 per year and much more if needed. Meanwhile, a woman in Sierra Leone can expect, on average, medicines worth about US$ 3 per year and, if she survives middle age and develop chronic diseases then she will die prematurely as a consequence of inadequate treatment. The contrasts in opportunities of treatment exist also within developing countries; between poor and rich, cities and rural areas and also between men and women.

In previous papers, the authors proposed mathematical models dealing with the burden of diabetes and its complications[3], Dynamics of a disabled population[4], the effect of physical exercise[5] and a model of dengue fever[6]. The present paper is devoted to the burden caused by NCDs in developing countries. In order to reverse the increasing trend of this burden (or at least to control it), the focus is made on the risk factors associated with these diseases.

Different methods can be considered to quantify the burden of NCDs. In order to overcome the specific problems of each country, the most used method is the approach that measures the global burden of NCDs in terms of Disability Adjusted Life Years (DALYs) which is a combination of Years of Life Lost(YLL) through premature death, and Years Lived with Disability(YLD). Thus, DALY is thought of as one lost year of healthy life [7-9]]. For example, deaths from underweight every year rob the world's poorest children of an estimated total of 130 million years of healthy life[10]. According to this approach, the burden of adult NCDs account for 80% in developed countries and for 70% in middle-income countries. Even in the high-mortality regions of the world, almost 50% of the adult disease burden is attributable to NCDs.

## Methods

Data from national registries and international sources are collected, compared and analyzed in order to show the trend of NCDs. Four diseases or cluster of diseases(Cardio-Vascular Diseases(CVDs), diabetes, cancers and chronic respiratory diseases) are considered to illustrate the growing burden of NCDs in developing countries. The main sources of data are the annual reports and regular publications released by the World Health Organization(WHO), World Heart Federation(WHF), Pan American Health Organization(PAHO), International Diabetes Federation (IDF), International Agency for Research on Cancer(IARC), Centre for Chronic Disease Prevention and Control(CCDPC), International Task Force for Prevention of Coronary Heart Disease and a multitude of websites and papers dealing with NCDs. The literature associated with these diseases in developed countries is abundant. However, despite the encouraging programmes and joint projects proposed by WHO and other organisms in the form of collaborative research agreements to developing countries, in order to support national registries, unreliable and insufficient data are still prevailing in most of these countries. Moreover, the release of health data is shadowed by the security vision in some countries. Extrapolations are needed in the case of missing or incomplete data. Consequently, more efforts are needed to convince health decision makers in low- and middle-income countries of the necessity to develop epidemiological studies that allow for preventive strategies making health policy at the centre of sustainable development.

## Results

According the World Health Organization's statistics, chronic NCDs such CVDs, diabetes, cancers, obesity and respiratory diseases, account for about 60% of the 56.5 million deaths each year and almost half of the global burden of disease. In 1990, 47% of all mortality related to NCDs was in developing countries, as was 85% of the global burden of disease and 86% of the DALYs attributable to CVDs. An increasing burden will be born mostly by these countries in the next two decades. The socio-economic transition and the ageing trend of population in developing countries will induce further demands and exacerbate the burden of NCDs in these countries. If the present trend is maintained, it is predicted that, by 2020, NCDs will account for about 70 percent of the global burden of disease, causing seven out of every 10 deaths in developing countries, compared with less than half today.

In 1990, approximately 1.3 billion DALYs were lost as a result of new cases of disease and injury, with the major part in developing countries. In 2002, these countries supported 80% of the global YLDs due to the double burden of communicable and non communicable diseases. Consequently, their people are not only facing higher risk of premature life(lower life expectancy) but also living a higher part of their life in poor health[1]. These remarks indicate that NCDs are exacerbating health inequities existing between developed and developing countries and also making the gap more profound between rich and poor within low and middle-income countries.

### CVDs in developing countries

CVD is the name for the group of disorders of the heart and blood vessels and include hypertension (high blood pressure), coronary heart disease (heart attack), cerebrovascular disease (stroke), peripheral vascular disease, heart failure, rheumatic heart disease, congenital heart disease and cardiomyopathies. These diseases constitute the major contributor among NCDs (Table 2).

**Table 2:** Deaths caused wordwide by specific diseases (× 10^3^)

Deaths & % Disease	2002 [1]	1990 [8]
Ischaemic heart disease	7000 (12.6%)	6260 (12.4%)
Cerebrovascular disease	5400 (09.6%)	4380 (08.7%)
Lower Respiratory Diseases	3700 (06.6%)	4300 (08.5%)
COPD	2700 (04.8%)	2211 (04.4%)
Cancer(all types)	7100 (12.6%)	6200 (11.2%)
Diabetes	3200 (05.6%)	2400 (05.0%)

Worldwide, an estimated 17 million people die of these diseases, particularly heart attacks and strokes, every year. Once associated with industrialized countries, CVDs are now emerging or rapidely increasing in developing countries. Indeed, in 1998, 86% of the DALYs caused by CVDs were attributed to developing countries and in 1999 CVDs contributed to a third of global deaths with 78% in low- and middle-income countries. The trend is increasing, indicating that by the year 2010 CVDs will be the leading cause of death in developing countries as a consequence of lifestyle changes brought about by industrialization and urbanization in developing countries engaged in the socio-economic transition. CVDs are promoted by risk factors like tobacco use, alcohol, physical inactivity and unhealthy diet. Unfortunately, the harm caused by these risk factors affects the rise of life expectancy in developing countries[1,11,12].

The costly and prolonged care of CVDs in low-and middle-income countries often divert the scarce family and societal resources to medical care. Consequently, the lower socio-economic groups have greater prevalence of risk factors, higher incidence of disease and higher mortality.

### Diabetes

The recent statistics released by the World Health Organization and the International Diabetes Federation are alarming[1,12](Table 3). The number of diabetes in the world is expected to increase from 194 Million in 2003 to 330 in 2030 with three in four living in developing countries. Moreover, in developed countries most people with diabetes are above the age of retirement, whereas in developing countries those most frequently affected are aged between 35 and 64 which makes the burden in terms of DALYs and YLDs heavier in poorer countries. Indeed, in some countries of the Middle East, one in four deaths in adults aged between 35 and 64 years is attributable to diabetes. The burden is exacerbated by the complications such as blindness, amputations and kidney failure for which diabetes is the leading cause, and the interfering action of CVDs which are responsible for between 50 and 80% of deaths in people with diabetes. The burden of premature death from diabetes is similar to that of HIV/AIDS, yet the problem is largely unrecognised [13].

**Table 3:** Diabetes prevalence (× 10^6^) [13]

Country	2000		2030
India	31.7	India	79.4
China	20.8	China	42.3
United States	17.7	United States	30.3
Indonesia	8.4	Indonesia	21.3
Japan	6.7	Pakistan	14.9
Pakistan	5.2	Bangladesh	11.8
Russia	4.6	Brazil	11.3
Brazil	4.5	Japan	8.9
Italy	4.2	Italy	5.4
Bengladesh	3.2	Russia	5.3

Studies in different countries have shown that diabetes is a costly disease accounting for between 2.5 and 15% of the total healthcare expenditure[3]. For the age category 20–79, the world annual direct cost is estimated to be over $153 billion and expected to double in 2025.

According to the National Institute of Diabetes and Digestive Kidney Disease(NIDDK) and the American Diabetes Association, diabetes was the sixth leading cause of death in 1999 with a direct cost of $44 billion and an indirect cost of $54 billion annually. In 2002, the direct and indirect cost totalled $132 billion.

In France, an estimation of $5.7 billion was given for the direct cost of diabetes, whereas, an equivalent cost of 5.2 billion, representing approximately 9% of the annual NHS budget, was given for UK in 2000.

The burden affects more and more developing countries as stressed by the different authors who attended the seventh congress of the Pan-African diabetes study group in 2001[14] and the Metabolic syndrome, type II diabetes, and atherosclerosis congress in 2004[15].

### Cancer

Cancer is now a major cause of mortality throughout the world (Table 4). In the developed world, it is generally exceeded only by CVDs but developing countries are responsible for the globally increasing trend. Over 10 million new cases and over 7 million deaths from cancer occurred worldwide in 2000[1,2,16-19]]. The contribution of developing countries was 53% for incidence and 56% for deaths (Table 5). From 1990 to 2000, the incidence and deaths increased by 2.4% per annum.

**Table 4:** Cancer by types and numbers worldwide (× 10^3^):

**Cancer**	**2000 [18] Incidence**	**%**	**2000 deaths**	**%**	**1990 Incidence**	**%**	**1990 [19] deaths**	**%**
Lung	1239	12.3	1103	17.8	1037	12.8	921	17.8
Breast	1050	10.4	373	6.0	796	9.8	314	6.1
Colorectal	945	9.4	492	8.0	783	9.7	437	8.4
Stomach	876	8.7	646	10.0	798	9.9	628	12.1
Liver	564	5.6	546	8.8	437	5.4	427	8.2
Prostate	543	5.4	204	3.3	396	4.9	165	3.2
Cervical	471	4.7	233	3.7	371	4.6	190	3.7
Oesophagus	413	4.1	337	5.4	316	3.9	286	5.5
Head&neck	390	3.9	207	3.3	306	3.8	162	3.1
Bladder	336	3.3	132	2.1	261	3.2	115	2.2
Other	3228	32.2	1934	31.0	2582	32.0	1537	30.0
Total	10055	100%	6209	53%	8083	100.0	5182	100.0

**Table 5:** Cancer in developing countries :incidence & deaths (× 10^3^) in 2000 [18]

**Cancer**	**Developing Incidence **	**%**	**Developing deaths**	**%**
Lung	792	14.7	522	14.6
Breast	471	8.8	184	5.6
Colorectal	334	6.2	252	7.0
Stomach	543	10.1	417	11.7
Liver	457	8.5	443	12.4
Prostate	127	2.4	76	2.1
Cervical	379	7.0	194	5.4
Oesophagus	341	6.3	274	7.7
Head&neck	262	4.9	154	4.3
Bladder	124	2.3	65	1.8
Other	1546	71.2	992	27.8
Total	5376	100%	3563	57.4

Between 2000 and 2020, the total number of cases of cancer in the developed world is predicted to increase by 29% whereas, in developing countries an increase by 73% is expected (largely as a result of an increase in the number of old people and as a result of urbanization and change in dietary habits).

The incidence of cancers of the lung, colon and rectum, breast and prostate generally increases in parallel with economic development, while the incidence of stomach cancer usually declines with development[2].

#### Lung cancer

This is currently the most common cancer in the world. In developed countries, smoking causes over 80% of such cancers and generally, heavy smoking increases the risk by around 30-fold making lung cancer a major problem in developing countries where the consumption of tobacco is flourishing.

#### Breast cancer

According to the International Agency for Research on Cancer (IARC), there were over a million new cases in the world in the year 2000, making it the second most common in the world and the most common among women with 47% in developing countries. Although rates are five times higher in industrialized countries, the burden of disease is heavier in poorer countries because breast cancer is highly curable if detected early and, unfortunately, about 80% of the cases are detected at advanced stages in developing countries.

#### Colorectal cancer

Ranking at the third place, with incidence rates tenfold higher in developed than in developing countries, this type of cancer is assumed to be mainly related to dietary factors which account to up to 80% of the between-country differences in rates.

#### Stomach cancer

20 years ago, this cancer used to be the most common in the world. At the moment, it is the fourth most common in the world but the second most common in developing countries. Substantial evidence suggests that risk is increased by high intakes of some traditionally preserved salted food and that risk is decreased by high intakes of fruit and vegetables.

#### Liver cancer

Approximately 75% of cases occur in developed countries, the rate vary over 20fold between countries. In developing countries, ingestion of contaminated food is an important risk factor together with active hepatitis virus infection whereas, alcohol consumption is the main diet-related risk factor in the world.

#### Cervical cancer

80% of the new cases and deaths are occurring in developing countries where it constitutes a major health problem. In developed countries, screening programmes and early detection have led to a noticeable decline in cervical cancer incidence and mortality, whereas, the trend is stable or increasing in low- and middle-income countries owing to their limited health care resources but also to their ill-health systems generating inefficient (or no)strategies[20].

#### Oral cavity, pharynx and oesophagus

In developed countries these types of cancer are mainly correlated to alcohol and tobacco(up to 75% of such cancers are attributable to these two lifestyle factors).

In developing countries, around 60% of such cancers are thought to be a result of micronutrient deficiencies related to a restricted diet that is low in fruit and vegetables and animal products. There is also consistent evidence that consuming drinks and foods at a very high temperature increases the risk for these cancers[2].

#### Pancreatic, endometrial, prostate and kidney cancers

These types of cancer are more common in industrialized countries. However, the fact that overweight/obesity is an established risk factor, their incidence is expected to increase in developing countries engaged in the socio-economic transition[2].

### Chronic respiratory diseases

Chronic respiratory diseases represent a major burden for the health systems worldwide. Most developing countries have no standard protocols for assessing and managing chronic non communicable respiratory diseases such as Chronic Obstructive Pulmonary Disease (COPD) and Asthma. In these countries, the population afflicted by poverty and illiteracy, having very little (or no) access to health services, will die before the age of 40 years. They comprise 15% of the population in Latin America, 34% in Arab world, 45% in Sub-Saharan Africa and south-east Asia[21,22].

Respiratory diseases cause 15% of the global burden of disease. Worldwide, it is estimated that 600 million people suffer from COPD and 2.5 million deaths were attributed to these diseases in 2000. By 2020, COPD is expected to become the third most common cause of mortality in the world.

## Discussion

### Risk factors: the enemies

In the previous sections, we considered four classes of non communicable diseases, namely, CVDs, diabetes, cancer and chronic respiratory diseases. Despite some differences between these classes and into each class, they do have a common denominator which is the risk factors. Indeed, Tobacco, alcohol, high blood pressure, diet and physical inactivity were indicated, at different levels, as risk factors in the four classes of NCDs. Moreover, these risk factors are seen to affect people worldwide with an increasing tendency. (Table 6)

**Table 6:** Burden of disease and risk factors worldwide:year 2002 [1]

Risk factor	Deaths (× 10^3^)	% of total death	DALYs (× 10^3^)	% of total DALY
Hypertension	7141	12.8	64270	04.5
Tobacco	4907	08.8	59081	04.1
High cholesterol	4415	07.9	40437	02.8
Low fruit & veg	2726	04.9	26662	01.9
Overweight	2591	04.6	33415	02.3
Alcohol	1804	03.2	58323	04.0
Phys. inactivity	1922	03.4	19092	01.3

Globally, many of the risk factors for heart disease, diabetes, cancer and pulmonary diseases are due to lifestyle and can be prevented. Physical inactivity, western diet and smoking are prominent causes[23]. Tobacco is the enemy number one. It is the most important established cause of cancer but also responsible in CVDs and chronic respiratory diseases. Tobacco and diet are the principal risk factors, responsible for more than 40% of cancer deaths and incidence. Obesity and dietary habits are the principal risk factors for diabetes of type 2.

### Tobacco[1,2,24]

In the 20 the century, approximately 100 million people died worldwide from tobacco-associated diseases such as cancers, chronic lung disease, diabetes and CVDs.

While tobacco consumption is falling in most developed countries, it is increasing in developing countries by about 3.4% per annum. Today, 80% of the 1.2 billion smokers in the world live in poorer countries where smoking prevalence among men is nearly 50% (48%) and 50% of the 5 million deaths attributed to smoking in 2000 occurred in developing countries, also responsible for the increase in deaths by more than one million during the last decade.

Tobacco remains the most important avoidable risk for the four classes of NCDs. It increases the risk of dying from coronary heart disease and cerebrovascular disease 2–3 fold. It increases the risk of many types of cancer, for lung cancer the risk is increased by 20–30fold. According to studies conducted in Europe, Japan and North America, 83–90% of lung cancers in men and 57–80 in women, are imputable to tobacco. Between 80 and 90 % of oesophagus, larynx and oral cavity are caused by tobacco and alcohol [17]. In developing countries, an estimated one-third of all cancer deaths was attributable to smoking in 1995.

Finally, tobacco exacerbates the conditions of people living with COPD and asthma.

### Lifestyle[2,25-27]

Up to 80% of cases of coronary heart disease, and up to 90% of cases of types 2 diabetes, could potentially be avoided through changing lifestyle factors.

One-third of cancers could be avoided by eating healthily, maintaining normal weight, and exercising throughout life.

It was estimated that in high-risk populations, an optimum fish consumption of 40–60 grams per day would lead to approximately a 50% reduction in death from coronary heart disease. A recent study based on data from 36 countries, reported that fish consumption is associated with a reduced risk of death from all causes as well as CVD mortality. Unfortunately, the fish consumption is very low even in some countries known for their large fish stock like the north African region.

Daily intake of fresh fruit and vegetables in adequate quantity (400–500 grams per day), is recommended to reduce the risk of coronary heart disease, stroke and high blood pressure. But, once more, this is thwarted by the western lifestyle invading developing countries.

### Overweight/Obesity[2,28]

Overweight and Obesity lead to adverse metabolic changes such as insulin resistance, increasing blood pressure and cholesterol. Consequently, they promote CVDs, diabetes and many types of cancer. Worldwide, overweight affects 1.2 billion of which 300 million are clinically obese. In some developed countries like USA, the prevalence reaches 60% but developing countries like Kuwait have also a very high prevalence. More and more children are suffering from overweight and obesity. However, the most contrasting phenomenon is to find Overweight/Obesity and malnutrition side by side in low- and middle-income countries and hence contributing to the growing burden afflicting these countries. According to the International Obesity Task Force (IOTF) and the WHO World Health report 2002, about 60% of diabetes globally can be attributable to overweight and obesity. In other respects, it is estimated that 60% of world's population do not do enough physical activity.

### Alcohol[2]

Alcohol consumption has also increased in the last decades, with the major part of this increase imputable to developing countries. In 2000, Alcohol was responsible for nearly 2 million deaths in the world, representing 4% of the global disease burden. Moreover, alcohol was estimated to cause 20 to 30 % of oesophagus cancer, liver disease, epilepsy, motor vehicle accidents and other hazards.

## Conclusions

Non communicable diseases are more and more prevalent in developing countries. These diseases are highly correlated to risk factors like smoking, alcohol, obesity, diet and inactivity. The World Health Organization and many other organisms and associations are urging health decision makers to develop efficient preventive strategies to halt the growing trend of NCDs through the control of risk factors. However, although most of developed countries have reacted by pragmatic measures, the trend remain globally passive mainly because developing countries have been, so far, satisfied with adopting national conventions and adhering to international recommendations instead of pragmatic decisions such as prohibiting smoking in public areas, controlling alcohol abusers, encouraging physical activity, promoting healthy diet and improving primary health care for screening and early detection of chronic diseases. In these countries, 2.8 billion people live with less than 2 dollars, 1.2 billion live with less than one dollar and 1.3 billion live on fragile and often remote rural ecosystems[29]. So, the behaviour can be partly explained by lack of means and poor budget affected to health care but, in general, bad management and absence of goodwill assume a large part of responsibility. For instance, many developing countries have signed the Framework Convention on Tobacco Control(FCTC) and voted laws that prohibits smoking in public areas but the laws are not executed. Also, in the absence of early detection, many people are diagnosed at advanced stages of cancer, cardiovascular diseases and diabetes complications. Also, in these countries, until recently, it was widely believed that economic development was a necessary prerequisite for improving a population health status and the health was often classified as a non productive sector. Now, politicians and health policy makers are timidly recognizing that investing in people's health is a necessary condition for economic development but energetic decisions are needed for the adoption of urgent and consequent strategies. The need for such strategies is enhanced by the fact that risk factors like cholesterol, tobacco, blood pressure, and obesity are no more a specificity of industrialized countries, they are becoming more prevalent in developing nations, where they double the burden of infectious diseases that have always afflicted poorer countries. Moreover, multinational companies have been competing fiercely to expand their sales in developing countries and western lifestyle is invading middle-income countries [2,30]. Adhesion to the Framework Convention on Tobacco Control(FCTC) and other international strategies must be taken seriously by developing countries facing the pandemics of NCDS.

## Competing interests

The author(s) declare that they have no competing interests.

## Authors' contributions

AB contributed by the collection of data concerning CVDs and diabetes and to English writing SB contributed by the collection of data concerning cancer and chronic pulmonary diseases.

The two authors contributed equally to the final version of the paper.
